# The Excess Polarizability of Single-Stranded DNA Molecules in Solution: A Linear Response Theory in the Polarizable Continuum Model with an Application to Biosensing

**DOI:** 10.1021/acs.jpca.5c03229

**Published:** 2025-07-25

**Authors:** Roberto Cammi

**Affiliations:** Dipartimento di Scienze Chimiche, della Vita e della Sostenibilitá Ambientale, 9370 Universitá degli Studi di Parma, Parco Area delle Scienze 11/a, Parma 43124, Italy

## Abstract

The excess polarizability of a molecular solute describes the interaction energy between a molecular solute and a macroscopic optical field that polarizes the solute itself, as compared to the same interaction process involving solvent molecules. The excess polarizability is involved in the physical mechanism that triggers the signals in the whispering gallery mode (WGM), a biosensing technique (denoted as WGM) for detecting biomolecules. The interpretation of the WGM signals requires information on the excess polarizability of the biomolecule of interest, which, until now, could be obtained from experimental measurements of the refractive index of solutions. Here, we present a quantum chemical theory for determining the excess polarizability using the effective linear response theory in the polarizable continuum model (PCM-eLRT) for solvation. As a proof-of-concept, the PCM-eLRT is applied at the TD-DFT level of electronic structure methodology to the calculation of the excess polarizability of single-stranded DNA molecules constituted up to 14-mer oligonucleotides, showing remarkable agreement with available experimental data.

## Graphical abstract

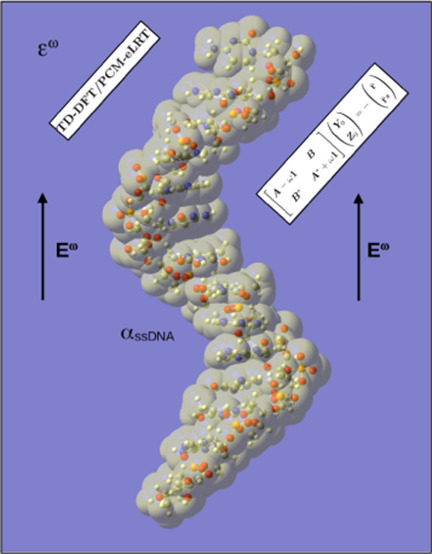

## 1 Introduction

Excess molecular polarizability in solutions has recently entered the common vocabulary of the molecular properties of interest in quantum chemistry, thanks to a recent intriguing observed optical phenomenon. The definition of the concept is simple: excess molecular polarizability is the electric dipole polarizability of a molecular solute, which is not taken in its absolute value but relative to the polarizability of the solvent molecules.^1–3^ The newly observed optical phenomena originating from the excess molecular polarizability are exploited in the whispering-gallery-mode biosensing method, WGM. In the WGM method, an optical radiation is made to circulate in a spherical microparticle immersed in a water solution containing a bioanalyte so that the circular resonance condition of the optical radiation is made sensible to the displacement of water molecules at the microparticle surface due to the binding of bioanalyte. The shift in the resonance condition is due to an evanescent tail of the optical field outside the microparticle, where it induces a dipole moment in the biomolecule in excess over the displaced water, causing a shift in the photon energy at the resonance.^2^

Theoretical and computational descriptions of the excess polarizability using classical or hybrid quantum-classical methods^4–6^ have been proposed, aiming to help decipher the signals of the WGM biosensor methodology. Theoretically, the excess polarizability is defined in terms of the so-called effective molecular polarizability of the solute and of the solvent (in our case, water).^5^ The effective molecular polarizabilities describe the induced dipole moment of the solute in response to a given macroscopic Maxwell field in the solvent and the corresponding interaction energy. The effective electric dipole polarizability of a molecular solute is a well-established concept in the classical Onsager cavity continuum model, which has several decades of applications.^4,7–13^ Within the Onsager model, the effective molecular polarizability is related to the polarizability of an isolated molecule using suitable classical factors that account both for the difference between the macroscopic field and the local field (the local field effect) and for the enhancing effect on the induced dipole moment due to the parallel interaction with surrounding molecules of the solvent (the reaction field effect). However, approaches based on the classical Onsager model must define the polarizability of an isolated molecule as a phenomenological parameter, as this property can only be determined by the laws of quantum mechanics.

Efficient and accurate calculation of the electric polarizability of isolated molecules can be performed from the quantum mechanical electronic structure methods using the linear response function theory.^14–17^ Furthermore, the effective polarizability of molecules in solution can be computed with the same efficiency and accuracy using the effective linear response theory of the polarizable continuum model,^18^ a quantum mechanical continuum solvation model.^19^

The effective linear response theory for molecules in solution, as described in the polarizable continuum model,^18,20–26^ is based on an accurate numerical solution of the local field problem for molecular-shaped (i.e., van der Waals) cavities, and allows the quantum mechanical calculation effective polarizability of the molecular solute in the presence of the effective perturbing fields of various nature (i.e., the local and reaction field contributions). In this paper, we extend the effective PCM-eLRT to the complete quantum-chemical calculation of the excess polarizability of molecular solutes of interest in the WGM biosensing method.

The paper is organized as follows. Section 2 presents the theoretical framework for defining and analyzing excess polarizability in the classical Onsager model and quantum mechanical PCM effective linear response theory. In Section 3, we present and discuss the numerical results of the PCM-eLRT method applied at the TD-DFT level of theory for the excess polarizability of selected single-strand oligonucleotides up to 14 oligomers. In Section 4, we conclude with some additional and final comments.

## 2 Theory

The theory section is separated into two subsections. In the first subsection, we present the derivation of the theory of excess polarizability according to the pure classical Onsager cavity field. This simple model has the advantage of transparently introducing the essential physical mechanisms that allow both the definition of the excess polarizability of the molecules in solution and the connection of the excess polarizability to the intrinsic molecular polarizability in the gas phase. The Onsager model is a phenomenological model, and the gas phase molecular polarizabilities can only be considered a parameter whose nature lies outside of the scope of the model.

In the second subsection, we present the theory of the excess polarizability in the polarizable continuum model.^19^ In this model, the excess polarizability of a molecular solute can be directly computed according to a full-quantum mechanical effective linear response function theory (eLRT). The PCM-eLRT is a generalization of the linear response theory for molecules in the gas phase,^14,15,17^ a generalization in which all the physical mechanisms that have been introduced by the Onsager model for the definition of excess polarizability are absorbed in the quantum mechanical formalism.^18,25^

### 2.1 The Excess Polarizability and the Effective Polarizabilities in the Onsager Classical Reaction Field Model

The Onsager reaction field model represents a molecular constituent of the solution (solute or solvent) as a polarizable point dipole (of intrinsic polarizability α and permanent dipole moment μ) located at the center of a spherical void cavity, of suitable radius *a*, cut out in the solvent. The solvent surrounding the molecular constituent is approximated as a uniform and homogeneous dielectric medium having the same frequency-dependent dielectric permittivity ϵ_ω_ of the pure solvent.^7,9,10^

#### 2.1.1 Effective Polarizability

Let us introduce in the medium the presence of a macroscopic alternative electric field of amplitude **E** ^ω^ (the Maxwell field) and associated with a monochromatic optical wave of frequency ω traveling in the solution.^27^ The Maxwell field is homogeneous far from a molecular cavity and satisfies the proper boundary condition at the molecular cavity surfaces.^9^ Inside the molecular cavity, the polarizable dipole experiences an effective local field $E_{L}^{\omega }$ that is different from the homogeneous Maxwell field and which induces an electric dipole moment δ**p** ^ω^ given by

1 $$\delta p_{J}^{\omega }=\alpha_{J}E_{L}^{\omega };,E_{L}^{\omega }=E_{c}^{\omega }+E_{\mathrm{RF}}^{\omega }$$

The cavity field amplitude $E_{c}^{\omega }\left(r\right)$ itself can be represented as the superposition of the homogeneous term, the Maxwell field far from the cavity (**E** ^ω^) and a second contribution $\left(E_{\Sigma }^{\omega }\left(r\right)\right)$ that originate by the boundary condition that the Maxwell field must satisfy at the cavity surface. For a spherical cavity, this second contribution is constant within the cavity and proportional to the same Maxwell field far from the cavity so that the cavity field can be written as

2 $$E_{c}^{\omega }=f_{c}^{\omega }E^{\omega };,f_{c}^{\omega }=\frac{3\epsilon^{\omega }}{2\epsilon^{\omega }+1}$$

where $f_{c}^{\omega }$ is called cavity field factor and ϵ^ω^ is the dielectric permittivity of the solvent at the frequency ω of the optical field that, according to the Maxwell relation, corresponds to the square of the refractive index, *n* ^ω^, of the solvent.

The reaction field amplitude $E_{\mathrm{RF}}^{\omega }$ is proportional to the induced dipole moment through the Onsager reaction field factor $f_{\mathrm{RF}}^{\omega }$

3 $$E_{\mathrm{RF}}^{\omega }=\delta p_{J}^{\omega }f_{\mathrm{RF}}^{\omega };,f_{\mathrm{RF}}^{\omega }=\frac{2\left(\epsilon^{\omega }-1\right)}{a^{3}\left(2\epsilon^{\omega }+1\right)}$$

where *a* is the cavity radius.

Combining eqs 1 [Disp-formula eq3] we can express the induced dipole moment in relation to the Maxwell field amplitude **E** ^ω^

4 $$\delta p_{J}^{\omega }={\mathrm{\alpha ̃}}_{J}^{\omega }E^{\omega }$$

with

5 $${\mathrm{\alpha ̃}}_{J}^{\omega }=f_{c}^{\omega }F_{\mathrm{RF}}^{\omega }\alpha_{J}$$

eq 4 is the phenomenological definition of ${\mathrm{\alpha ̃}}_{J}^{\omega }$ as the effective polarizability of the molecule with respect to the homogeneous macroscopic Maxwell optical field. Thus, according to eq 5 in the Onsager model the effective polarizability differs from the polarizability of the isolated molecule by a factor $f_{c}^{\omega }$ which translates the macroscopic field to a cavity field and by a factor $F_{\mathrm{RF}}^{\omega }=\frac{1}{1-f_{\mathrm{RF}}^{\omega }\alpha }$ which accounts of the enhancing effect (reaction field effect) of the surrounding dielectric on the induced dipole moment.

#### Energy-Effective Polarizability, ${\mathrm{\alpha ̅}}_{J}^{\omega }$

2.1.2

We now consider the problem of evaluating the energy of the induced moment $\delta p_{J}^{\omega }$ in relation to the macroscopic Maxwell optical field **E** ^ω^. This relation can be deduced starting from the expression of the energy of the induced moment in the cavity field $E_{c}^{\omega }$ :

6 $$W\left(\delta p_{J}^{\omega };E^{\omega }\right)=-\frac{1}{2}\delta p_{J}^{\omega }\cdot E_{c}^{\omega }$$

Introducing eqs 2 and 4 into eq 6, we can express the interaction energy in relation to the macroscopic optical field **E** ^ω^ as

7 $$W\left(\delta p_{J}^{\omega };E^{\omega }\right)=-\frac{1}{2}{\mathrm{\alpha ̅}}_{J}^{\omega }E^{\omega }E^{\omega }$$

with ${\mathrm{\alpha ̅}}_{J}^{\omega }$ given by

8 $${\mathrm{\alpha ̅}}_{J}^{\omega }=f_{c}^{\omega }{\mathrm{\alpha ̃}}_{J}^{\omega }$$

or

9 $${\mathrm{\alpha ̅}}_{J}^{\omega }={\left(f_{c}^{\omega }\right)}^{2}F_{\mathrm{RF}}^{\omega }\alpha_{J}$$

eq 7 defines the *energy-effective* polarizability ${\mathrm{\alpha ̅}}_{J}^{\omega }$ of the molecular solute that describes the effective energy of the induced dipole relative to the Maxwell optical field.^28^ According to eqs 8 and 9, ${\mathrm{\alpha ̅}}_{J}^{\omega }$ differs from the effective polarizability ${\mathrm{\alpha ̃}}_{\omega }$ by the cavity field factor $f_{c}^{\omega }$ and as a consequence ${\mathrm{\alpha ̅}}_{J}^{\omega }$ differs from the polarizability of the isolated molecule by the square of the cavity field factor $f_{c}^{\omega }$ times the reaction field factor $F_{\mathrm{RF}}^{\omega }$ . Expressions for the *energy-effective* polarizability ${\mathrm{\alpha ̅}}_{J}^{\omega }$ has been previously derived by Buhmann and co-workers^4^ and by Zossimova et al.^5^

#### Excess Polarizability, ${\mathrm{\alpha ̅}}_{B|A}^{\omega }$

2.1.3

The *energy-effective* polarizability ${\mathrm{\alpha ̅}}_{J}^{\omega }$ for both the solute and the solvent have a central role in defining the excess polarizability of a solute that triggers the signals in the WGM experiments.^2^ The excess polarizability describes the difference between the energy of the induced dipole moment of a biomolecular solute (say B) and the corresponding energy for the induced dipole moments of the number of water molecules (say A) that occupy the same volume of the biomolecule.^5^ This energy difference is given by

10 $$W_{B|A}\left(E^{\omega }\right)=W\left(\delta p_{B}^{\omega };E^{\omega }\right)-W\left(\delta p_{A}^{\omega };E^{\omega }\right)\frac{V_{B,m}}{V_{A,m}}$$

where $W\left(\delta p_{B}^{\omega };E^{\omega }\right),W\left(\delta p_{A}^{\omega };E^{\omega }\right)$ are, respectively, the energy of the polarized biomolecule and water in relation to the optical Maxwell field, and *V*_m, A_, *V*_m, B_ are the molar volumes of the biomolecule and water, respectively.

Introducing eq 7 into eq 10 we obtain the energy difference $W_{B|A}^{\mathrm{WGM}}$ expressed as

11 $$W_{B|A}\left(E^{\omega }\right)=-\frac{1}{2}{\mathrm{\alpha ̅}}_{B|A}^{\omega }E^{\omega }E^{\omega }$$

with

12 $${\mathrm{\alpha ̅}}_{B|A}^{\omega }={\mathrm{\alpha ̅}}_{B}^{\omega }-{\mathrm{\alpha ̅}}_{A}^{\omega }\frac{V_{B,m}}{V_{A,m}}$$

Equation 12 defines the excess polarizability ${\mathrm{\alpha ̅}}_{B|A}^{\omega }$ of the solute B with respect to the solvent A. As we have said in the introduction, the excess molecular polarizability of a bioanalyte plays a key role in the biosensing WGM methods. In fact, according to the analytical theory of Arnold et al.,^2^ in a WGM measurement, the relative shift of the resonance condition of the radiation with a microsphere is proportional to the excess polarizability of the bioanalyte that binds at the surface of the microsphere.

Introducing eq 9 into eq 8, the excess polarizability in the Onsager model can be determined as

13 $${\mathrm{\alpha ̅}}_{B|A}^{\omega }={\left(f_{c}^{\omega }\right)}^{2}F_{\mathrm{RF},A}^{\omega }\alpha_{B}^{\omega }-{\left(f_{c}^{\omega }\right)}^{2}F_{\mathrm{RF},B}^{\omega }\alpha_{A}^{\omega }\frac{V_{B,m}}{V_{A,m}}$$

This equation gives a crystal clear account of the physical mechanisms that, in the Onsager model, translate the polarizability of the isolated molecules A and B into the energy-effective excess polarizability in a solution of B in A. Furthermore, this equation has an operative value for small to medium-sized molecules with a shape such that the spherical Onsager cavity model may be a reasonable approximation of the molecules in solution. In these cases, the dynamical polarizabilities $\alpha_{A}^{\omega },\alpha_{B}^{\omega }$ of the isolate molecules can be computed using the linear response theory,^14,15,17^ as implemented in the computational quantum chemistry packages, and eq 13 can be exploited to estimate the excess polarizability.^4–6^

### 2.2 The Polarizable Continuum Model and the Excess Polarizability from a Quantum Chemistry Linear Response Theory

The polarizable continuum model^19,29^ is an ab initio quantum chemical method aimed at describing the properties of a target molecular system while it interacts electrostatically with the solvent represented as a dielectric medium having the same dielectric properties as the pure solvent. The molecular solute occupies a void cavity carved in the dielectric medium with a shape and size determined by the solute’s geometry and by the space accessible only to the nuclei and electrons of the solvated molecules. The simplest cavity that satisfies this requirement is a van der Waals molecular cavity.^30^

#### 2.2.1 The Quantum Mechanical Properties of Solvated Molecules

The molecular quantum problem is thus coupled with a classical electrostatic problem of determining the polarization of the dielectric medium under the electrostatic potential of the charged particles (electrons and nuclei) and the electrostatic potential that the polarized medium acts back on the charged particle (the reaction field). The electrostatic problem requires the solution of the electrostatic Poisson equation for the entire system, molecule, and dielectric with the given boundary condition at the surface of the molecular-shaped cavity. In PCM, the Poisson equation is solved numerically by exploiting boundary element methodologies (BEM)^31,32^ that represent the reaction field potential in terms of a discrete set of point charges (called apparent polarization charges) spread on the cavity surface.^33^ The electronic Hamiltonian operator of the molecular solute is thus obtained by adding to the Hamiltonian of the isolated molecules terms representing the interaction of electrons and nuclei with the polarization charges. These terms contain contributions with one-particle and pseudotwo-particle natures, so the PCM Hamiltonian can be written in terms of one- and two-particle contributions.^19^

The effective electronic Hamiltonian for the molecular solute in a given electronic state wave function Ψ can be written as^19^

14 $$H\left(\Psi \right)=H^{o}+\langle \Psi \left|Q_{e+n}^{\mathrm{BEM}}\right|\Psi \rangle \cdot V_{e+n}$$

where *H* ^o^ is the electronic Hamiltonian of the isolated molecule Ψ is the electronic wave function and $Q_{e+n}^{\mathrm{BEM}}$ is a vector operator determining the apparent polarization charges of the solvent at the cavity surface for a given Cartesian configuration the electrons (e) and nuclei itself (n) and **V**_e+n_ is a vector collecting the molecular electrostatic potential operator (electrons (e) and nuclei (n)) at the cavity surface on the position of the apparent polarization charges.

Approximate solutions of the quantum problem for the solvated molecule can be obtained at the same level as the standard quantum chemical methods for isolated molecules. We do not give details, for which one may refer to the abundant literature.^19^ Suffice here to say that there is a variational principle for the electronic energy associated with the electronic Hamiltonian of the solute and that all the time-independent molecular properties of the solvated molecule can be computed as the expectation value of the related operator or as a suitable derivative of the electronic energy with respect the amplitude of an external perturbation described in terms of the same operator representing the property of interest.^25^

For example, in the case of the density functional theory, the Kohn–Sham matrix operator that determines the K–S molecular orbitals of the molecular solute in a suitable expansion basis set has elements given by

15 $$f_{\mu \nu }^{\mathrm{PCM}}=\left(h_{\mu \nu }+j_{\mu \nu }\right)+\underset{\lambda \sigma }{\sum }P_{\lambda \sigma }^{\mathrm{KS}}\left[\left\langle \mu \lambda \right|\left|\nu \sigma \right\rangle +B_{\mu \nu ,\lambda \sigma }\right]$$

where *h*_μν_ are the matrix elements, in the AO basis, of the one-electron core operator, ⟨μλ||νσ⟩ are the antisymmetrized combination of regular two-electron repulsion integrals (ERIs) and $P_{\mu \nu }^{\mathrm{HF}}$ indicate the elements of the Hartree–Fock density matrix in the AO basis. The matrix elements *j*_μν_, $B_{\mu \nu ,\lambda \sigma }$ are the one- and two-electron type integrals that map the solute–solvent operator of eq 14 in the space spanned by the AO basis set.^19^

The DFT electric dipole moment of a molecular solute is then determined as

16 $$\left\langle \mu \right\rangle =\underset{\lambda \sigma }{\sum }P_{\lambda \sigma }^{\mathrm{KS}}\mu_{\lambda \sigma }$$

where μ_μν_ are the matrix elements of the electric dipole vector operator **μ** in the AO basis set.

#### 2.2.2 The Effective Interaction of a Molecular Solute with a Maxwell Optical Field

Consider a molecule in the solution with a macroscopic optical Maxwell field of amplitude **E** ^ω^ and frequency ω traveling. The field is homogeneous far from the molecular vdW cavity and it satisfies the usual boundary condition of the electric field at the boundary of molecular cavity.^34^ The electronic Hamiltonian for the molecular solute is obtained by adding to eq 14 a term describing the effective interaction with the optical Maxwell field. This contribution can be expressed in terms of an effective cavity scalar potential $V\left(E_{c}^{\omega }\right)$ corresponding to the amplitude of the cavity electric field $E_{c}^{\omega }$ inside the molecular vdW cavity. The problem of determining $V\left(E_{c}^{\omega }\right)$ is a generalization to the more general molecular vdW cavities of the cavity field problem we met in the Onsager model for a spherical cavity.^18,20,25,35,36^

The effective cavity scalar potential $V\left(E_{c}^{\omega }\right)$ due to the optical Maxwell field **E** ^ω^ can be written as^18,35^

17 $$V(r;E_{c}^{\omega }=V\left(r;\;E^{\omega }\right)+V\left(E_{\Sigma }^{\omega }\left(r\right)\right)$$

where *V*(**r**;**E** ^ω^) is the electrostatic potential due to the Maxwell field far from the molecular cavity, and $V\left(E_{\Sigma }^{\omega }\left(r\right)\right)$ is the contribution originated by the boundary condition that the Maxwell field must satisfies at the boundary of the molecular vdW cavity. We have demonstrated^18^ that this contribution can be determined for a general vdW cavity by using a numerical Boundary Elements Method approach so that the total scalar potential at a general position **r** can be written in terms of an effective and explicit dependence from the Maxwell field **E** ^ω^

18 $$V\left(r;E_{c}^{\omega }\right)=r\cdot E^{\omega }+{\mathrm{r̃}}_{\Sigma }\left(r\right)\cdot E^{\omega }$$

where the first term on the right side represents the contribution of the homogeneous Maxwell field far from the molecular cavity, and the second term is the contribution due to the boundary condition on the Maxwell field, with **r̃**(**r**) given by a suitable position depending vector that it is related to the jump of the Maxwell field at the boundary of the vdW cavity.^37^

Introducing eqs 18 and 19 into eq 14 we obtain an effective time-dependent Hamiltonian of the molecular solute in relation to the macroscopic Maxwell field **E** ^ω^(*t*):

19 $$H\left(t\right)=H^{0}+\langle \Psi \left(t\right)\left|Q_{e+n}^{\mathrm{BEM}}\right|\Psi \left(t\right)\rangle \cdot V_{e+n}-\mathrm{\mu ̅}\cdot E^{\omega }\left(e^{-i\omega t}+e^{+i\omega t}\right)$$

where Ψ­(*t*) is the time-dependent wave function of the molecular solute and **μ̅** is an effective electric dipole moment operator. **μ̅** is given by adding to the bare electric dipole moment operator **μ** an operator **μ̃**_**Σ**_ representing the presence of the boundary conditions of **E** ^ω^(*t*) at the vdW molecular cavity:

20 $$\mathrm{\mu ̅}\left(\left\{r_{i}\right\}\right)=\mu +{\mathrm{\mu ̃}}_{\Sigma }$$

with **μ** and μ̃_Σ_ given by

21 $$\mu =-\overset{N}{\underset{i}{\sum }}r_{i};,{\mathrm{\mu ̃}}_{\Sigma }=-\overset{N}{\underset{i}{\sum }}{\mathrm{r̃}}_{\Sigma }\left(r_{i}\right)$$

where **r**_**i**_ denotes the Cartesian-coordinate of electron *i* and is the number of electrons.

The time evolution of the wave function Ψ­(*t*) is driven by the time-dependent effective Hamiltonian (21) according to the related time-dependent Schrödinger equation.^38^ Associated with this time-dependent Schrödinger equation, there is a time-dependent variational principle^38^ and a time-dependent quasi-energy variational principle.^25,39^ Thus, approximate solutions of the time-dependent Schrödinger equation for the electronic Hamiltonian of the solute can be obtained at the same levels of theory as the standard quantum chemical methods for isolated molecules. Furthermore, an effective time-dependent linear response theory for the effective Hamiltonian (eq 21) describes the polarizabilities we previously defined within the Onsager model.^18,25^

#### 2.2.3 The Effective Polarizabilities in the QM/PCM Linear Response Theory

The effective linear response function of the quantum mechanical polarizable continuum model (PCM-eLRT)^25^ is a generalization of the response function theory for isolated molecules^14^ to the case of a molecule interacting with a polarizable medium representing the solvent. The essence of the PCM-eRFT is thus to characterize the time evolution of the expectation value of a property *A* of a molecular solute due to the interaction with the optical Maxwell field as described by the time-dependent operator *V*(*t*) of eq 21.

The time evolution of the observables of interest A (i.e., the electric dipole moment) due to effective perturbation with the Maxwell field is expanded in a series of the amplitude of the Maxwell field **E** ^ω^, and at the first order we have

22 $$\langle \Psi \left(t\right)\left|A\right|\Psi \left(t\right)\rangle =\langle \Psi \left(-\infty \right)\left|A\right|\Psi \left(-\infty \right)\rangle +\langle \langle A;\mathrm{\mu ̅}\rangle \rangle \cdot E^{\omega }\left(e^{-i\omega t}+e^{+i\omega t}\right)$$

where $\langle \langle A;\mathrm{\mu ̅}{\rangle \rangle }_{\omega }$ is the effective linear response function. $\langle \langle A;\mathrm{\mu ̅}{\rangle \rangle }_{\omega }$ describes the Fourier component of the time-dependent response of the property *A* of the molecular solute due to the presence of the optical monochromatic macroscopic Maxwell field *E* ^ω^(*t*).

In the language of the linear response functions [Disp-formula eq24], we can easily find the quantum mechanical definition of effective and energy-effective polarizabilities of a molecular solute, which have been introduced phenomenologically in eqs 4 and 7, respectively, for the classical Onsager model.

- The effective polarizability ${\mathrm{\alpha ̃}}_{J}^{\omega }$

  According to eq 4, the effective polarizability ${\mathrm{\alpha ̃}}_{J}^{\omega }$ describes the induced electric dipole moment in relation to the optical Maxwell field. Thus, it corresponds to the linear response function for the electric dipole moment operator *A* = **μ**_J_:
23 $${\mathrm{\alpha ̃}}_{J}^{\omega }=-\langle \langle \mu_{J};{\mathrm{\mu ̅}}_{J}{\rangle \rangle }_{\omega }$$
  The effective polarizability ${\mathrm{\alpha ̃}}_{J}^{\omega }$ (eq 24) can be directly compared with experimental data from the refractive index of pure solvents or solutions (see Supporting Information).^10,11,18,23,26^

- The energy-effective polarizability ${\mathrm{\alpha ̅}}_{J}^{\omega }$

  As defined in eq 7, the energy-effective polarizability describes the energy of the induced dipole moment in relation to the optical Maxwell field. This energy can be expressed in terms of the expectation value of the effective molecule-field interaction operator of eq 19:
24 $$W\left(\delta p_{J}^{\omega };E^{\omega }\right)=\frac{1}{2}E^{\omega }\cdot \langle \langle {\mathrm{\mu ̅}}_{J};{\mathrm{\mu ̅}}_{J}{\rangle \rangle }_{\omega }\cdot E^{\omega }$$
  Therefore, energy-effective polarizability corresponds to the linear response function for the effective dipole operator *A* = ***μ̅***_J_
25 $${\mathrm{\alpha ̅}}_{J}^{\omega }=-\langle \langle {\mathrm{\mu ̅}}_{J};{\mathrm{\mu ̅}}_{J}{\rangle \rangle }_{\omega }$$
  As described in the Supporting Information, also the energy-effective polarizability ${\mathrm{\alpha ̅}}_{J}^{\omega }$ can be compared with experimental data from measurements of the refractive index of pure solvents or solutions.
  The difference between the energy-effective and effective polarizability is related to a cavity field effect, and, in analogy with eq 8 of the Onsager model, the ratio between ${\mathrm{\alpha ̅}}_{J}^{\omega }$ and ${\mathrm{\alpha ̃}}_{J}^{\omega }$ defines an effective cavity field factor for the vdW cavity of the PCM model:^20^
26 $$f_{c,\mathrm{vdW}}^{\omega }=\frac{{\mathrm{\alpha ̅}}_{J}^{\omega }}{{\mathrm{\alpha ̃}}_{J}^{\omega }}$$
  In the limit of a spherical molecular cavity, the value of effective cavity field factor *f*_c,vdW_ reduces to that of the Onsager cavity field factor of eq 2.
- Noneffective polarizability ${\mathrm{\alpha ̅}}_{J}^{\omega }$

  Finally, another type of polarizability that can be defined is the noneffective polarizability $\alpha_{J}^{\omega }$ . The noneffective polarizability corresponds to the polarizability of the molecular solute when the interaction operator of the molecule with the Maxwell field neglects the effect of the boundary conditions on the Maxwell field itself.^5,40^ The noneffective polarizability corresponds to the linear response function:
27 $$\alpha_{J}^{\omega }=-\langle \langle \mu_{J};\mu_{J}\rangle \rangle \omega$$
- Excess polarizability of a solute (B) in a solvent (A) ${\mathrm{\alpha ̅}}_{B|A}^{\omega }$

  Introducing eq 27 into eq 12, the excess polarizability of a solute can be expressed in terms of the energy-effective linear response functions of the solute and solvent as
28 $${\mathrm{\alpha ̅}}_{B|A}^{\omega }=-\langle \langle {\mathrm{\mu ̅}}_{B};{\mathrm{\mu ̅}}_{B}{\rangle \rangle }_{\omega }+\langle \langle {\mathrm{\mu ̅}}_{A};{\mathrm{\mu ̅}}_{A}{\rangle \rangle }_{\omega }\frac{V_{B,m}}{V_{A,m}}$$

Explicit expressions of the effective and noneffective polarizabilities have been derived for the various levels of the electronic structure methods.^18,25,39,40^ These expressions can take the form of a sum over the excited states of the molecular solute (the so-called Lehmann representation). However, as in the case of isolated molecules,^14^ all the polarizabilities can be computed without recourse to explicit sum-overstate (SOS) calculations. The explicit expressions for the PCM-eLRT polarizabilities in the TD-DFT approximation are reported in the Supporting Information (eqs B.1–B.10).

## 3 TD-DFT/PCM-eLRT Excess Polarizability of ss-Dna Molecules

This section focuses on the TD-DFT/PCM-eLRT calculations of the excess polarizability ${\mathrm{\alpha ̅}}_{J|w}^{\omega }$ of single-strand DNA molecules (J) in water (w). The section is divided into three subsections. The first and second subsections present and analyze the results of the effective polarizability ${\mathrm{\alpha ̅}}_{w/J}^{\omega }$ of single molecules, water, and ss-DNA, respectively. In the third subsection, we present and discuss the results of the computed excess polarizability of the ss-DNA systems ${\mathrm{\alpha ̅}}_{J|w}^{\omega }$ . This discussion compares the TD-DFT/PCM-eLRT results with the available experimental data from measurements of the refractive index of DNA solutions.^3,41^

### 3.1 Water: The Effective Polarizabilities

The effective and energy-effective polarizabilities, ${\mathrm{\alpha ̅}}_{w}^{\omega },{\mathrm{\alpha ̃}}_{w}^{\omega }$ , of water have been computed at the TD-DFT^42,43^ level using the *wB*97*XD* hybrid exchange-correlation functional^44^ and the *aug-cc-pvqz* ^45–49^ basis set and at a frequency ω corresponding to an optical Maxwell field of 1312.8 nm. The calculations refer to the equilibrium geometry of solvated water (w), which has been computed at the DFT^50^ level using the *wB*97*XD* hybrid exchange-correlation functional and the *cc-pvqz* ^48^ expansion basis set. All the calculations have been performed for solvated water with Gaussian 16 ^51^ using the IEF-PCM,^31^ with default parameters for the vdW cavity and the solvent dielectric static dielectric permittivity ϵ = 78.355300 and frequency-dependent dielectric permittivity at 1312.8 nm ϵ^ω^ = 1.7485.^52^

The values of the TD-DFT/PCM-eLRT isotropic effective polarizabilities α̅_w,iso_, α̃_w,iso_ of water are shown in Table 1. In the same Table we report the experimental values of α̅_w,iso_, α̃_w,iso_. We first comment on the increase in values when passing from effective and energy-effective polarizability, and then on the comparison with the experimental data. The increases in the polarizability in passing from α̃_w,iso_ to α̅_w,iso_ is due to the cavity field effect (as expected from eq 5). The corresponding ratio $\frac{{\mathrm{\alpha ̃}}_{w,\mathrm{iso}}}{{\mathrm{\alpha ̅}}_{w,\mathrm{iso}}}$ defines, according to eq 26 an effective cavity field factor for the vdW PCM cavity *f*_c,w_ = 1.1536.^20^

**Table 1:** Comparison between the Values of the Computed Effective, α̃_w,iso_, and Energy-Effective, α̅_w,iso_, Isotropic Polarizabilities of Water and the Corresponding Experimental Values ${\mathrm{\alpha ̃}}_{w,\mathrm{iso}}^{\exp}$ and ${\mathrm{\alpha ̅}}_{w,\mathrm{iso}}^{\exp}$ , Respectively (See Text for Details)^a^

α̃_w,iso_	α̅_w,iso_	${\mathrm{\alpha ̃}}_{w,\mathrm{iso}}^{\exp}$	${\mathrm{\alpha ̅}}_{w,\mathrm{iso}}^{\exp}$
1.7367	2.0035	1.7871	2.0612

a All the values are in Å^3^.

The experimental value of the effective polarizability, ${\mathrm{\alpha ̃}}_{w,\mathrm{iso}}^{\exp}$ , has been determined, according to eq A.5 in the Supporting Information, from the value of the refractive *n*_w_ = 1.3223 and molar volume *V*_w,m_ = 18.069 cm^3^/mol at 25 °C.^54^ The experimental data of the energy-effective polarizability, ${\mathrm{\alpha ̅}}_{w,\mathrm{iso}}^{\exp}$ , has been estimated according to eq A.6 in the Supporting Information by scaling the experimental value of ${\mathrm{\alpha ̃}}_{w,\mathrm{iso}}^{\exp}$ by the effective cavity scaling factor *f*_w,c_ = 1.1536. The computed and experimental values of the effective polarizabilities differ only by 2.8%. A similar quantitative agreement between calculated and experimental effective polarizability has been observed in other pure liquids and solutions.^23,24,26^

### 3.2 ss-DNA: Energy-Effective Polarizability

We considered two sets of ss-DNA molecules corresponding to 14 oligomer sequences. Their sequences, reported in Table 2, were constructed from two distinct oligonucleotides, 11-mer and 14-mer, which have been investigated in a whispering-gallery-mode (WGM) experiment of DNA quantification by Vollmer et al.^3^ The initial geometries of the ss-DNA systems have been obtained from the website.^53^ Subsequent geometry optimizations were performed using the self-consistent semiempirical DFT tight-binding method xtb. xtb calculations incorporated a generalized Born solvation model and water as solvent.^57,58^ The xtb tight-binding DFT method has proven to be an efficient approach to obtain reliable structures of biopolymers, including DNA.^59–61^

**Table 2:** Sequences of the Single-Stranded DNA Molecules Considered in This Work^a^

ss-DNA (J)
5′-CT-3′
5′-CTA-3′
5′-CTATC-3′
5′-CTATCT-3′
5′-CTATCTCA-3′
5′-CTATCTCAG-3′
5′-CTATCTCAGTC-3′
5′-TAT-3′
5′-TATGA-3′
5′-TATGAA-3′
5′-TATGAATT-3′
5′-TATGAATTC-3′
5′-TATGAATTCAAT-3′
5′-TATGAATTCAATCC-3′

a Nucleic acids are denoted with their canonical labels, cytosine (C), guanine (G), adenine (A), and thymine (T), and are shown from left to right in the 5′ to 3′ order.

#### 3.2.1 Effect of the Basis Set on the Energy-Effective Polarizability

The TD-DFT/PCM-eLRT calculations of the effective polarizabilities of all the ss-DNA molecules have been performed for the optical Maxwell field of 1312.8 nm with the Gaussian 16 software package^51^ and using the same *wB*97*XD* ^44^ hybrid functional used for the water molecule, but a basis set different from that used for water has been employed. Benchmark study on the effect of the basis set on the electric dipole polarizability in the gas phase using DFT methods has shown the need for diffuse functions to obtain a reasonable accuracy in the value of the dipole polarizability.^62^ Hence, we first studied the effect of the basis set on the effective polarizabilities for the shortest sequences of each ss-DNA system, 5′-CT-3′, 5′-TAT-3′, considering the basis sets *aug*-*cc*-*pvdz* and the *cc* -*pvdz*. From the results of these calculations, which are reported in Table 3, the effect of the addition of diffuse function has been approximated as a scaling factor of 1.1751 that we have applied as a pragmatic account of the effect of the diffuse functions to the effective polarizabilities of all the other longer sequences of DNA molecules, that have been computed with the *cc* - *pvdz* basis set. The scaled *cc* -*pvdz* results will be denoted as (*aug*)-*cc*-*pvdz*.

**Table 3:** PCM-eLRT/wB97XD Values (Å^3^) of Isotropic Energy-Effective Polarizability α̅_w,iso_ of the 5′-CT-3′ and 5′-TAT-3′ Oligonucleotides in Water and at 1312.8 nm^a^ ^b^

ss-DNA	cc-pvdz	aug-cc-pvdz	$\frac{\mathrm{aug‐cc‐pvdz}}{\mathrm{cc‐pvdz}}$
5′-CT-3′	58.0	68.4	1.1799
5′-TAT-3′	93.4	109.4	1.1703

a The second and third columns report the values for the cc-pvdz and aug-cc-pvdz basis sets, respectively.

b The last column reports the ratio value between the outcomes of two basis sets.

#### 3.2.2 Correlation of Energy-Effective Polarizability with the Molar Mass

The TD-DFT/PCM-eLRT-(aug)-cc-pvdz values of the energy-effective isotropic polarizabilities of the ss-DNA molecules are reported in Table 4 as a function of the molecular mass values *M*_J_ of the molecules. Their correlation is shown in Figure 1. A notable feature is that the energy-effective isotropic polarizability correlates linearly with the molar mass: with a linear coefficient 0.1253 ± 0.000 Å^3^/(gr/mol), a constant 2.04 ± 0.31 Å^3^ and a *R*-*square* 1.00. This phenomenological correlation is a manifestation of an effective property of additivity of the polarizability of the chemical units components of the single-stranded DNA molecules.

**Table 4:** Values (Å^3^) of the TD-DFT/PCM-eLRT/wB97XD/(aug)­cc-pvdz Energy-Effective Polarizability in Water and at 1312.8 nm as a Function of the Molar Mass (gr/mol) of the ss-DNA Molecules

ss-DNA	α̅_J,is*o*_	*M*_J_
5′-CT-3′	68.4	530.4
5′-CTA-3′	108.1	842.6
5′-CTATC-3′	181.5	1434.0
5′-CTATCT-3′	219.1	1737.1
5′-CTATCTCA-3′	295.2	2337.5
5′-CTATCTCAG-3′	335.9	2665.7
5′-CTATCTCAGTC-3′	409.0	3257.1
5′-TAT-3′	109.4	857.6
5′-TATGA-3′	190.2	1498.0
5′-TATGAA-3′	229.2	1810.2
5′-TATGAATT-3′	305.2	2416.6
5′-TATGAATTC-3′	341.0	2704.8
5′-TATGAATTCAAT-3′	458.0	3632.3
5′-TATGAATTCAATCC-3′	529.8	4208.7

**Figure 1:**
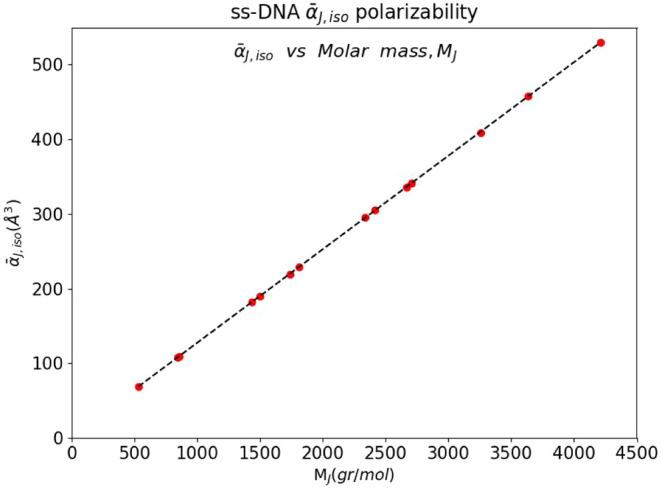
Correlation between the computed values the energy-excess polarizability, α̅_J,iso_ [Å^3^], and the molar mass, *M*_J_ [gr/mol], of ss-DNA molecules (see text for details).

### ss-DNA: Excess Polarizability ${\mathrm{\alpha ̅}}_{J|w}^{\omega }$

3.3

The TD-DFT/PCM-eLRT excess polarizability of the ss-DNA molecules has been computed according to eq 28. According to this equation, the excess polarizability is determined by the energy-effective polarizability of the solute minus the energy-effective polarizability of the solvent times a factor given by the ratio between the molar volume of the solute and the solvent. We have used the values of the energy-effective polarizability of the ss-DNA sequences from Table 4 (wB97XD/(aug)-cc-pvdz) and the values of the energy-effective polarizability of water computed at the wB97XD/aug-cc-pvqz level (see Table 1). The ratio between the molar volumes of the ss-DNA and water has been approximated as the corresponding ratio between volumes of the vdW-PCM cavities[Fn ba-fn1]. The values of the TD-DFT/PCM-eLRT excess polarizabilities of the ss-DNA molecules are reported in Table 5.

**Table 5:** Excess Polarizability (Å^3^) of the ss-DNA Molecules in Water: Comparison between the TD-DFT/PCM-eLRT Computed, α̅_J|w_, and Experimental, ${\mathrm{\alpha ̅}}_{J|w}^{\mathrm{Exp}.}$ , Values (See Text for Details)

ss-DNA	${\mathrm{\alpha ̅}}_{J|w}^{\mathrm{PCM}}$	${\mathrm{\alpha ̅}}_{J|w}^{\mathrm{Exp}.}$
5′-CT-3′	35.5	36.1
5′-CTA-3′	58.3	57.5
5′-CTATC-3′	99.0	98.3
5′-CTATCT-3′	119.6	119.2
5′-CTATCTCA-3′	161.9	160.7
5′-CTATCTCAG-3′	185.4	183.4
5′-CTATCTCAGTC-3′	225.7	224.3
5′-TAT-3′	58.6	58.6
5′-TATGA-3′	105.0	102.8
5′-TATGAA-3′	127.1	124.6
5′-TATGAATT-3′	169.3	166.4
5′-TATGAATTC-3′	188.9	186.2
5′-TATGAATTCAAT-3′	254.0	2450.6
5′-TATGAATTCAATCC-3′	293.4	290.3

Table 5 also reports the experimental values of the excess polarizability of the DNA molecules. The experimental values of ${\mathrm{\alpha ̅}}_{B|A}^{\omega }$ have been computed according to eq A.10 in the Supporting Information. According to this equation, the experimental excess polarizability of a DNA molecule is determined by the refractive index *n* of pure water, the refractive index coefficient of the DNA solution as a function of the mass concentration of DNA, $\frac{dn_{\omega }}{dm_{B}}$ , the molar mass of DNA, *M*_J_ and by the effective cavity field factor $f_{c}^{\omega }$ . We have used the value *n* = 1.332 and $\frac{dn_{\omega }}{dm_{B}}=0.168\;{\mathrm{cm}}^{3}/\mathrm{gr}$ for the refractive index of water and the refractive index of the DNA solution $\frac{dn_{\omega }}{dm_{B}}=0.168\;{\mathrm{cm}}^{3}/\mathrm{gr}$ , respectively.^41^ The values of the molar mass of DNA are taken from Table 4, and the effective cavity field factor values $f_{c}^{\omega }$ have been computed according to eq 26 and reported in the Supporting Information.

Figure 2 illustrates the comparison between the theoretical predictions TD-DFT/PCM-eLRT and the experimental data. This figure reveals a surprising agreement between the two. They are in a very strong linear correlation: with a linear coefficient 1.0128 ± 0.003, a constant −0.268 ± 0.492 Å^3^ and a *R*-*square* 1.000.

**Figure 2:**
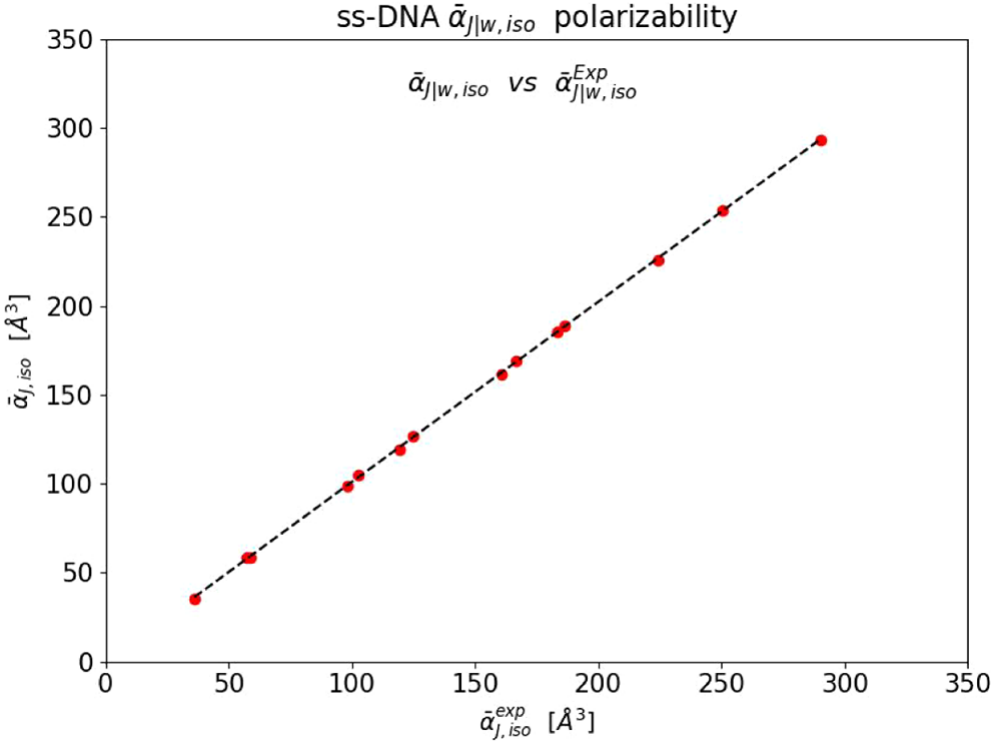
Correlation between the values of the computed ${\mathrm{\alpha ̅}}_{J|w}^{\mathrm{PCM}}$ and experimental ${\mathrm{\alpha ̅}}_{J|w}^{\mathrm{Exp}.}$ isotropic excess polarizability (Å^3^) of the ss-DNA molecules in water (see text for details).

We can not escape commenting on this agreement between theory and experiment. Surely, caution must be assumed, and other applications of the TD-DFT/PCM-eLRT theory will be required to provide a more robust confirmation of this agreement. However, granting some recognition to this agreement, a pertinent question emerges: since the theory TD-DFT/PCM-eLRT merges an electronic structure theory (TD-DFT) and a continuum solvation theory (PCM-eLRT), which cooperate to compute the excess polarizability, what is the contribution of each of these theories to the overall agreement?

Surely, the accuracy of the TD-DFT method plays a crucial role in computing the dipole polarizability of molecular systems. Using a hybrid exchange-correlation energy functional along with a split-valence basis set that includes polarization and diffuse functions can yield accurate values for the dipole polarizability of molecules such as the basis of DNA.^63,64^ However, it must recognize the merit of a coherent integration of the TD-DFT methods within the effective linear response theory of the PCM model (PCM-eLRT), which enables us to address the problem of the interaction between a molecular solute and the optical macroscopic Maxwell field, which is a parameter under control in the experiment.

We finally note that, as suggested by one of the reviewers, even in the context of the apparent agreement with experimental data of the present work, one must bear in mind the well-known general limitations of the implicit solvation model, like PCM, such as the lack of description of specific solute–solvent interactions. These interactions can be more accurately described by QM solvation models that incorporate an explicit (i.e., atomistic) description of the solvent. The specific solute–solvent interactions can affect the spectroscopic properties of molecular solutes, as recently discussed by Giovannini and Cappelli,^65^ and it is foreseeable that in the not-too-distant future it will be possible to calculate excess polarizations even by explicit solvation methods.

## 4 Conclusions

We have presented an electronic structure method for calculating the excess polarizability of molecules in solution. The method has been applied to the calculation of single-strand DNA molecules. The numerical results compare well with the available experimental data of the excess polarizability of ss-DNA molecules. This agreement may suggest that the TD-DFT/PCM-eLRT computational method could become a practical support in deciphering the whispering-gallery-mode (WGM) biosensing experiments of ss-DNA molecules. Indeed, further confirmation and validation of TD-DFT/PCM-eLRT through computational studies of other ss-DNA systems will be necessary.

From a methodological perspective, we should not notice that some aspects of the TD-DFT/PCM-eLRT could be improved or expanded. At the QM level, the TD-DFT level can give accurate polarizabilities with enough accuracy. However, it may not become feasible with DNA systems much larger than those considered in the present study. The PCM-eLRT theory could be extended to the tight-binding TD-DFT methods if a suitable parametrization for calculating the electric dipole polarizability is provided. We have also seen that the calculation of excess polarizability depends on the ratio of the molar volumes of bioanalytes to water, and we have approximated this ratio in terms of the ratio of the corresponding vdW cavity volumes. However, other choices of molecular cavities, such as SAS cavities^66^ or SES cavities,^67^ are possible and deserve consideration in future studies.

## Supplementary Material





## Data Availability

The data that supports the findings of this study are available within the article and its Supporting Information.

## Footnotes

a The values of the vdW-PCM cavities are reported in the Supporting Information.

## Notes

ssDNA geometries (ZIP)
Geometry optimizations of the ss-DNA molecules; experimental measurement of the excess polarizabilities from refractive index of solutions; TD-DFT/PCM-eLRT equations; volumes of the vdW-PCM cavities; dependence of the excess polarizability on the cavity scaling factor, *f*, of PCM (PDF)
