# Supplementary material for: The Excess Polarizability of Single-Stranded DNA Molecules in Solution: A Linear Response Theory in the Polarizable Continuum Model with an Application to Biosensing
Source: J Phys Chem A. 2025 Jul 25;129(31):7130–9. doi: 10.1021/acs.jpca.5c03229 (PMC12337146; doi:10.1021/acs.jpca.5c03229)
Supplement: Supplementary file 2 [file jp5c03229_si_003.pdf]

The excess polarizability of single-stranded DNA molecules in solution: a linear response theory in the Polarizable Continuum Model with an application to biosensing.

Roberto Cammi,*
Dipartimento di Scienze Chimiche, della Vita e della Sostenibilitá Ambientale,
Università degli Studi di Parma, Parco Area delle Scienze 11/a, 43124 Parma, Italy
e-mail: roberto.cammi@unipr.it

Contents

I. **Geometry optimizations of the ss-DNA molecules** 2

II. **Experimental measurement of the excess polarizabilities from refractive index of solutions** 3

III. **TD-DFT/PCM-eLRT equations** 5

IV. **Volumes of the vdW-PCM cavities** 7

V. **Effective cavity field factors f_c^{PCM}** 9

VI. **Dependence of the excess polarizability on the cavity scaling factor, f, of PCM** 10

References 11

References 11

I. GEOMETRY OPTIMIZATIONS OF THE SS-DNA MOLECULES

For all the ss-DNA sequences reported in Table II of the main text, the geometry optimizations have been performed using the self-consistent semi-empirical DFT tight-binding [2, 3] incorporating a generalized Born solvation model and using water as the solvent [1]. Geometry optimizations were performed using a two-step procedure for the ss-DNA molecules up to six nucleotides. In the first step, an optimization has been performed with a level convergence on the gradient norm of $5 * 10^{-3} Eh/a_0$ (level loose). The geometries were then reoptimized using the level of convergence with gradient norm of $1 * 10^{-3} Eh/a_0$) (level normal). The stationary points were characterized through the Hessian calculations. For all the larger ss-DNA sequences, geometry optimizations were performed with a level convergence on the gradient norm of $1 * 10^{-3} Eh/a_0$ (level loose). The Cartesian coordinates of the optimized geometries are reported in the attached compressed file.

II. EXPERIMENTAL MEASUREMENT OF THE EXCESS POLARIZABILITIES FROM REFRACTIVE INDEX OF SOLUTIONS

In this appendix, we review how the effective and energy-effective optical molecular polarizability $\widetilde{\alpha}_J, \bar{\alpha}_J$ can be determined from measurements of the refractive index n of a solution as a function of the concentrations, according to Ref.s [5, 6].

The refractive index n_ω of a solution is related to the macroscopic optical linear susceptibility $\chi_\omega^{(1)}$ of the solution as

$$\chi_\omega^{(1)} = \frac{n_\omega^2 - 1}{4\pi} \tag{A.1}$$

Assuming additivity of the molecular contributions to the macroscopic polarization, the optical linear susceptibility $\chi_\omega^{(1)}$ of can related to the concentrations $c_J(mol * cm^{-3})$ of the components of the solution

$$\chi_\omega^{(1)} = \sum_J \zeta_{J,\omega}^{(1)} c_J \tag{A.2}$$

where the quantities $\zeta_{J,\omega}^{(1)}(mol^{-1}cm^3)$ are the molar polarizabilities given by

$$\zeta_{J,\omega}^{(1)} = N_A \widetilde{\alpha}_J^\omega \tag{A.3}$$

where N_A is the Avogadro constant and $\widetilde{\alpha}_{J,\omega}^\omega$ are the experimental effective polarizability of the molecular constituents. Hence, by introducing Eq.s (A.1-2) into Eq. (A.3) we obtain the following relation between the refractive index of a solution and the effective polarizability of the molecular constituents:

$$\frac{n_\omega^2 - 1}{4\pi} = N_A \sum_J \widetilde{\alpha}_J^\omega c_J \tag{A.4}$$

In the case of a pure solvent (e.g., A), we obtain the effective polarizability of the solvent molecules in terms of the refractive index of the pure solvent:

$$\widetilde{\alpha}_{A,\omega} = \frac{n_{A,\omega}^2 - 1}{4\pi} \frac{V_{A,m}}{N_A} \tag{A.5}$$

where $V_{A,m}$ if the molar volume of the solvent, A. This equation allows to determine experimentally the effective polarizability $\widetilde{\alpha}_{A,\omega}$ from the measured refractive index, and in turn

$\widetilde{\alpha}^{exp}_{A,\omega}$ that can be compared with the effective polarizability computed according to Eq. (23) of the PCM-eLRT theory, $\widetilde{\alpha}^{PCM}_{A,\omega}$.

The energy-effective molecular polarizability $\bar{\alpha}^{\omega}_{A}$ of the solvent may estimated from the effective molecular polarizability $\widetilde{\alpha}^{\omega}_{A}$ as

$$\bar{\alpha}^{\omega}_{A} = f^{\omega}_{c}\widetilde{\alpha}^{\omega}_{A} \tag{A.6}$$

where f^{ω}_{c} is the cavity field factor estimated according to the Onsager model (Eq. 8) of the PCM-eLRT theory (see Eq.(26)).

In the case of a dilute solution of B in the solvent A. Eq. (A.4) leads to a linear relation between the square of the refractive index n^2 of the solution and the concentration of B

$$\frac{n^2_{\omega} - 1}{4\pi N_A} = \frac{n^2_{A,\omega} - 1}{4\pi N_A} + \widetilde{\alpha}^{\omega}_{B|A} c_B \tag{A.7}$$

where $\widetilde{\alpha}^{\omega}_{B|A}$ is the effective excess polarizability of the solute B in the solvent A:

$$\widetilde{\alpha}^{\omega}_{B|A} = \left(\widetilde{\alpha}_{B,\omega} - \widetilde{\alpha}_{A,\omega}\frac{V_{B,m}}{V_{A,m}}\right) \tag{A.8}$$

Differentiating Eq. (A.7) with respect to the concentration c_B we obtain the following expression for the experimental effective excess polarizability $\widetilde{\alpha}^{\omega}_{B|A}$

$$\widetilde{\alpha}^{\omega}_{B|A} = \frac{2n}{4\pi N_A}\frac{dn_{\omega}}{dc_B} \tag{A.9}$$

where $\frac{dn_{\omega}}{dc_B}$ is the derivative of the refractive index n_{ω} with respect to the concentration mol/cm^3 of the solute. A similar equation has been previously proposed by Vollmer [7]. The experimental energy-effective excess polarizability $\bar{\alpha}^{\omega}_{B|A}$ can be obtained as

$$\bar{\alpha}^{\omega}_{B|A} = f^{\omega}_{c}\frac{2n}{4\pi}\frac{dn_{\omega}}{dm_B}\frac{M_B}{N_A} \tag{A.10}$$

where f^{ω}_{c} is the cavity field factor that should be estimated according to the Onsager model (Eq. 8) of the PCM-eLRT theory (see Eq. (26)), $m_B(gr/cm^3)$ is the mass concentration of the solute and $M_B(gr/mol)$ its molar mass. The need of a local field factor correction in the evaluation of the experimental excess polarizability has been previously recognized by

Teraok and Arnold [8].

In this paper, we will consider the experimental excessive polarizability $\bar{\alpha}^{\omega}_{B|A}$ of DNA molecules according to the experimental data of Ref.[9], with a refractive index $n = 1.332$ a value of $\frac{dn_\omega}{dm_B} = 0.168 \quad cm^3/gr$, and the effective cavity field factor f^{ω}_c computed according to Eq. (26).

III. TD-DFT/PCM-ELRT EQUATIONS

This appendix reviews the PCM-eLRT matrix equations for the TD-HF/KS cases. Analogous expressions for the TD-MCSCF and TD-CCSD cases can be found in Ref.s [10–12]. For the case of an electronic state described by a single determinant wavefunction, the effective linear response functions describing the properties of a molecular solute in relation to a perturbation due to a monochromatic Maxwell optical field can be written as [10]

$$<< O; \bar{\boldsymbol{\mu}} >>_\omega = \begin{pmatrix} \boldsymbol{o} \\ \boldsymbol{o}^* \end{pmatrix}^\dagger \begin{bmatrix} \boldsymbol{A} - \omega \mathbf{1} & \boldsymbol{B} \\ \boldsymbol{B}^* & \boldsymbol{A}^* + \omega \mathbf{1} \end{bmatrix}^{-1} \begin{pmatrix} \bar{\mathbf{r}} \\ \bar{\mathbf{r}}* \end{pmatrix} \tag{B.1}$$

Here, $O = \sum_i^N o(i)$ is the operator of the property of interest (e.g. $\boldsymbol{\mu}$ or $\hat{\boldsymbol{\mu}}$ of Eq.s ( )); $\boldsymbol{o}$ is a vector of length given by the number of occupied spin-orbitals N_{occ} times the number of vacant spin-orbitals N_{virt} collecting the integrals of the operator $o(i)$ among the unoccupied and occupied KS/HF molecular orbitals (the so-called particle-hole space):

$$o_{ai} = \langle a|o|i\rangle \tag{B.2}$$

where we have used the convention of denoting with $i, j, ..$ occupied and with $a, b, ..$ vacant spin-orbitals. Similarly, $\bar{\mathbf{r}}$ is a vector of length $N_{occ}N_{virt}$ collecting the integrals of the effective dipole operator $\bar{r}_\alpha(i)$ among the unoccupied and occupied KS/HF molecular orbitals:

$$\bar{r}_{ai} = \langle a|\bar{r}|i\rangle \tag{B.3}$$

Square matrices $\boldsymbol{A}, \boldsymbol{B}$ have dimension $N_{occ}N_{virt}$ and elements:

$$A_{ai,bj} = \delta_{ai,bj}(\epsilon_a - \epsilon_i) + \langle aj||ib\rangle + \mathcal{B}_{ai,bj} \tag{B.4}$$

$$B_{ai,bj} = \langle ab||ij\rangle + \mathcal{B}^{\omega}_{ai,bj} \tag{B.5}$$

where $\langle ab||ij\rangle$ are the usual two-electron integrals in the basis of the occupied and vacant spin-orbitals, and $\mathcal{B}^{\omega}_{ai,bj}$ is an effective two-electron integral related to the reaction field originated by the electrons. To be more specific, $\mathcal{B}^{\omega}_{ai,bj}$ describe an electrostatic electron-electron interaction mediated by the external medium (electron 1 polarizes the dielectric whose reaction field acts back on electron 2). The upper script ω of $\mathcal{B}^{\omega}_{ai,bj}$ denotes that the reaction field is evaluated using the value of the dielectric permittivity ϵ_ω of the solvent at the same frequency as the optical Maxwell field.

The frequency dependent linear response function $<< O;\bar{\boldsymbol{\mu}} >>_\omega$ has poles $\omega_f$in correspondence of the eigenvalues of the generalized eigenvalue problem

$$\begin{bmatrix} \boldsymbol{A}-\omega\mathbf{1} & \boldsymbol{B} \\ \boldsymbol{B}^* & \boldsymbol{A}^*+\omega\mathbf{1} \end{bmatrix} \begin{pmatrix} \mathbf{Y}_f \\ \mathbf{Z}_f \end{pmatrix} = \omega_f \begin{bmatrix} \mathbf{1} & \mathbf{0} \\ \mathbf{0} & -\mathbf{1} \end{bmatrix} \begin{pmatrix} \mathbf{Y}_f \\ \mathbf{Z}_f \end{pmatrix} \tag{B.6}$$

Poles ω_f and the corresponding residues of the linear response function are the approximations of excited energy and transition moments of the molecular solute. The effective linear response function can then be formally expressed as a summation over all the excited states from Eq. (B.6). However, the linear response function can be evaluated by avoiding the complete solution of this eigenvalue problem . Instead, the linear response function can be evaluated as:

$$<< O;\bar{\boldsymbol{\mu}} >>_\omega = \begin{pmatrix} \boldsymbol{o}^\dagger & \boldsymbol{o}^{*\dagger} \end{pmatrix} \begin{pmatrix} \boldsymbol{Y}_0 \\ \boldsymbol{Z}_0 \end{pmatrix} \tag{B.7}$$

where $\begin{pmatrix} \boldsymbol{Y}_0 \\ \boldsymbol{Z}_0 \end{pmatrix}$ is the solution of the simple linear system of equations:

$$\begin{bmatrix} \boldsymbol{A}-\omega\mathbf{1} & \boldsymbol{B} \\ \boldsymbol{B}^* & \boldsymbol{A}^*+\omega\mathbf{1} \end{bmatrix} \begin{pmatrix} \boldsymbol{Y}_0 \\ \boldsymbol{Z}_0 \end{pmatrix} = - \begin{pmatrix} \bar{\mathbf{r}} \\ \bar{\mathbf{r}}* \end{pmatrix} \tag{B.8}$$

IV. VOLUMES OF THE VDW-PCM CAVITIES

The vdW-PCM cavities of water and the ss-DNA molecules are generated by the vdW spheres centered on the constituting atoms as shown in Figure C.1. The reference radii of the atomic vdW sphere $R_H = 1.443\mathring{A}, R_C = 1.9255\mathring{A}, R_N = 1.83\mathring{A}, R_O = 1.75\mathring{A}, R_P = 2.0735\mathring{A}$, are scaled by a common factor $f = 1.1$. [4]. The following table reports the volume of the resulting vdW-PCM cavities of all the molecular systems.

TABLE C.1: Values of the volume $V_{J,c}[\mathring{A}^3]$ of the vdW-PCM cavities of water and ss-DNA molecules.

System	$V_{J,c}$
Water	37.3
5'-CT-3'	612.9
5'-CTA-3'	928.7
5'-CTATC-3'	1538.4
5'-CTATCT-3'	1853.8
5'-CTATCTCA-3'	2484.8
5'-CTATCTCAG-3'	2803.4
5'-CTATCTCAGTC-3'	3416.5
5'-TAT-3'	946.3
5'-TATGA-3'	1587.9
5'-TATGAA-3'	1902.1
5'-TATGAATT-3'	2532.2
5'-TATGAATTC-3'	2834.1
5'-TATGAATTCAAT-3'	3800.5
5'-TATGAATTCAATCC-3'	4404.2

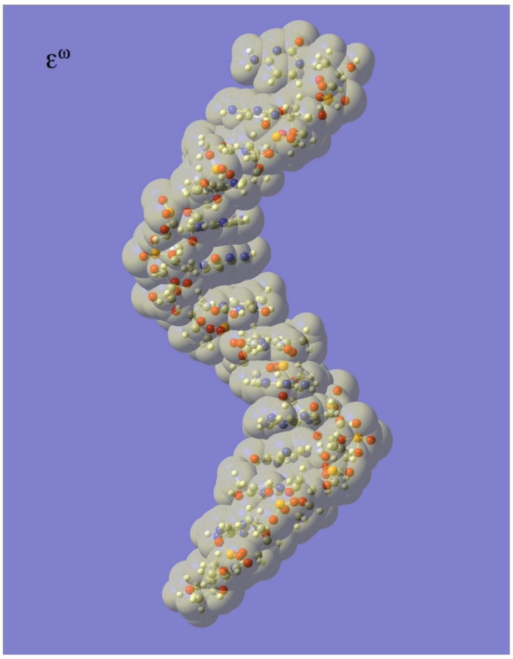

FIG. C.1: The vdW-PCM cavity of the 14-mer oligonucleotide DNA in a dielectric medium of permittivity ϵ^ω

V. EFFECTIVE CAVITY FIELD FACTORS f_c^{PCM}

Table D.1 reports the values of the effective cavity field factors f_c^{PCM} of the ss-DNA molecules. The effective cavity field factors f_c^{PCM} have been computed according to Eq. (26) of the main text, from the effective polarizabilities $\tilde{\alpha}_{J,iso}, \bar{\alpha}_{J,iso}$ of the ss-DNA molecules in the presence of a macroscopic Maxwell field $\mathbf{E}^{\omega}$ (see Figure D.1).

TABLE D.1: Values of cavity field factors f_c^{PCM} of the ss-DNA molecules.

ss-DNA	f_c^{PCM}
5'-CT-3'	1.1520
5'-CTA-3'	1.1546
5'-CTATC-3'	1.1589
5'-CTATCT-3'	1.1607
5'-CTATCTCA-3'	1.1624
5'-CTATCTCAG-3'	1.1631
5'-CTATCTCAGTC-3'	1.1642
5'-TAT-3'	1.1556
5'-TATGA-3'	1.1607
5'-TATGAA-3'	1.1635
5'-TATGAATT-3'	1.1645
5'-TATGAATTC-3'	1.1641
5'-TATGAATTCAAT-3'	1.1663
5'-TATGAATTCAATCC-3'	1.1661

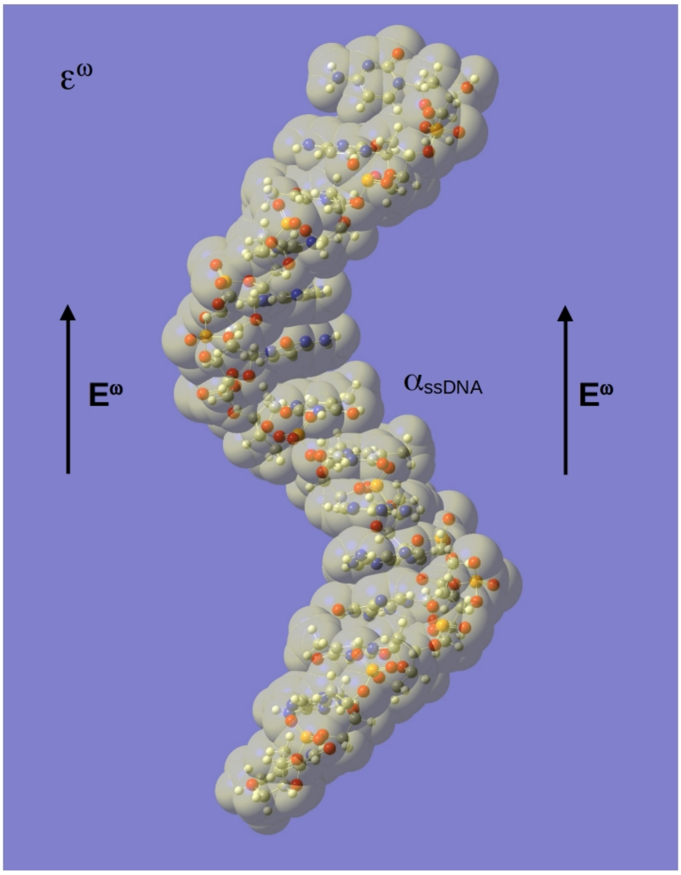

FIG. D.1: The 14-mer oligonucleotide DNA into a vdW-PCM cavity in the presence of an optical Maxwell field within a dielectric medium of permittivity ϵ^{ω}. The PCM-eLRT theory determines the effective polarizabilities $\tilde{\alpha}_{J,iso}, \bar{\alpha}_{J,iso}$ of the DNA molecule in relation to the Maxwell field, explicitly accounting of the jump of the field at the boundary of the vdW cavity

.

VI. DEPENDENCE OF THE EXCESS POLARIZABILITY ON THE CAVITY SCALING FACTOR, F, OF PCM

As a preliminary test of the dependence of the value of the excess polarizability on slightly variations of PCM cavity scaling factor,f, for two selected systems(5'-CT-3' , 5'-TAT-3') are reported in Table D.2. These results demonstrate the satisfactory robustness of the computed excess polarizability with respect to this parameter of the PCM model: we can

observe a variation of less than one percent (the technical error bar) for a reasonable slightly variation of the cavity scaling factor f value compared to the default value (f=1.1) of Gaussian 16 [4].

TABLE D.2: Values of excess polarizability of two ss-DNA molecules as a function of the choice of the cavity scaling factor f in the PCM-eLRT calculations. The values of the excess polarizability are in $\mathring{A}^3$. The cavity scaling factor f is varied about the default value $f = 1.1$ of PCM in Gaussian 16.

	f				
ss-DNA	1.00	1.05	1.10	1.15	1.20
5'-CT-3'	35.41	35.50	35.52	35.56	35.46
5'-TAT-3'	58.39	58.50	58.57	58.70	58.48

References

[1] In all the sequences, the total charge of the ss-DNA molecules is equal to the number of nucleotides in the sequence minus one.

[2] Grimme, S., Banwarth, G., Shushkov, P., *J. Chem. Theory Comp.*, **2017**,*13*, 1989-2009.

[3] Banwarth, G., Ehlert, S., Grimme, S., *J. Chem. Theory Comp.*, **2019**, *15*, 1652-1671.

[4] Gaussian 16, Revision B.01, M. J. Frisch, G. W. Trucks, H. B. Schlegel, G. E. Scuseria, M. A. Robb, J. R. Cheeseman, G. Scalmani, V. Barone, G. A. Petersson, H. Nakatsuji, X. Li, M. Caricato, A. V. Marenich, J. Bloino, B. G. Janesko, R. Gomperts, B. Mennucci, H. P. Hratchian, J. V. Ortiz, A. F. Izmaylov, J. L. Sonnenberg, D. Williams-Young, F. Ding, F. Lipparini, F. Egidi, J. Goings, B. Peng, A. Petrone, T. Henderson, D. Ranasinghe, V. G. Zakrzewski, J. Gao, N. Rega, G. Zheng, W. Liang, M. Hada, M. Ehara, K. Toyota, R. Fukuda, J. Hasegawa, M. Ishida, T. Nakajima, Y. Honda, O. Kitao, H. Nakai, T. Vreven, K. Throssell, J. A. Montgomery, Jr., J. E. Peralta, F. Ogliaro, M. J. Bearpark, J. J. Heyd, E. N. Brothers, K. N. Kudin, V. N. Staroverov, T. A. Keith, R. Kobayashi, J. Normand, K. Raghavachari, A. P. Rendell, J. C. Burant, S. S. Iyengar, J. Tomasi, M. Cossi, J. M. Millam, M. Klene, C.

Adamo, R. Cammi, J. W. Ochterski, R. L. Martin, K. Morokuma, O. Farkas, J. B. Foresman, and D. J. Fox, Gaussian, Inc., Wallingford CT, 2016.

[5] Liptay, W., Becker,J., Wehing, D., Lang, W., Burkhard, O., The determination of molecular quantities from measurements on macroscopic systems. II the determination of electric dipole moments., *Z. Naturforsch.* **1982***37a*, 1396-1408.

[6] Wolfe. J.J, and Wortmann, R., Organic materials for second-order non-linear optics, in *Adv. Phys. Org. Chem.*, **1999**,*32*, 121-217.

[7] Vollmer, F., Arnold, S., Braun, D., Teraoka, I., Libchaber, A., Multiplexed DNA quantification by spectroscopic shift of two microsphere cavities *Biophys. J.* **2002**, *85*, 1973.

[8] Teraoka, I., Arnold, S. , Theory of resonance shifts in TE and TM wihspering gallery modes by nonradial perturbations for sensing applications., *Op. Soc. of Am.* , **2006**,*B23*, 1381.

[9] Nicolai, T., Van Dijk, L, Van Dijk, J.P.A., Smit, J.A.M., Molecular mass characterization of DNA fragments by gel permeation chromatography using a low-angle laser light scattering detector. *J. Chromatogr.*, **1987**,*389*, 286-292.

[10] Cammi, R., Mennucci, B., Linear response theory for the polarizable continuum model, *J. Chem. Phys.*, **1999** *110*, 9877-9886.

[11] Cammi, R., Frediani, L., Mennucci, B., Ruud, K., *J. Chem. Phys.*, Multiconfigutational self-consistent field linear response for the polarizable continuum model: theory and application to ground and excited state polarizabilities of para-nitroaniline in solution, **2003** *119*, 5818-5827.

[12] Cammi, R. *Molecular Response Functions for the Polarizable Continuum Model: Physical basis and quantum mechanical formalism*, Springer, Heidelberg, **2013**.
